# Evidence of *Anopheles stephensi* involvement in the transmission of *Plasmodium vivax* in Djibouti City using highly sensitive sporozoite detection assay

**DOI:** 10.1186/s13071-026-07532-9

**Published:** 2026-07-08

**Authors:** Subhika Rao, Jeanne N. Samake, Cristina Rafferty, Peter Mumba, Sheleme Chibsa, Meshesha Balkew, Bouh Abdi Khaireh, Samatar Kayad Guelleh, Mohamed Mousse Ibrahim, Abdoulilah Ahmed Abdi, Sarah Zohdy

**Affiliations:** 1 U.S. Centers for Disease Control and Prevention, National Center for Emerging and Zoonotic Infectious Diseases, Entomology Branch, Atlanta, GA USA; 2 Akros Bridge the Gap Malaria Initiative, Lusaka, Zambia; 3 Former staff of Abt Global PMI Evolve Ethiopia Project, Addis Ababa, Ethiopia; 4 Association Mutualis, Djibouti City, Djibouti; 5 Global Fund Program Management Unit, OGPP, Ministry of Health, Djibouti, Djibouti; 6 Programme National de Lutte Contre le Paludisme, Ministère de la Santé, Djibouti City, Djibouti; 7 National Public Health Institute, Djibouti City, Djibouti; 8 Health Council of the Presidency of the Republic of Djibouti, Djibouti City, Djibouti; 9 Service de Santé des Armées, Présidence de la République, Djibouti City, Djibouti

**Keywords:** *Anopheles stephensi*, Invasive species, Malaria transmission, *Plasmodium vivax*

## Abstract

**Background and Objective:**

*Anopheles stephensi* is a malaria mosquito vector that has been raising international concern due to its invasive nature in Africa, including the nation of Djibouti. Since its initial detection in Djibouti in 2012, malaria morbidity and mortality have increased exponentially in the country. With the observed increases in human malaria cases and previously reported An. stephensi involvement in outbreaks, high-quality evidence of Plasmodium sporozoite-carrying *An.* *stephensi* is essential to confirm and monitor the role of the invasive vector in malaria transmission dynamics in Djibouti. This study aims to detect Plasmodium sporozoites in *An. stephensi* collected in Djibouti using a multiplex circumsporozoite ELISA bead assay and assess the genetic relatedness of these populations to *An. stephensi* across the Horn of Africa.

**Methods:**

One hundred and ninety-six wild caught adult *An. stephensi* mosquitoes from Djibouti City were collected, morphologically identified, and molecularly confirmed using a portion of the cytochrome c oxidase subunit 1 marker. All samples were then tested for infective sporozoites *Plasmodium vivax 210, P. vivax 247 and P. falciparum* using a multiplex circumsporozoite enzyme-linked immunosorbent assay bead assay. One hundred and fourteen COI sequences that passed further sequence analysis quality control for comparative population genetic analysis were used to characterize the genetic diversity of the sampled *An. stephensi* and its genetic relatedness to other *An. stephensi* populations across the Horn of Africa.

**Results:**

All 196 samples were morphologically and molecularly confirmed to be *An. stephensi.* Plasmodium vivax 210 sporozoites were detected in two specimens with a positivity rate of 1.0%. No Pv247 or Pf sporozoites were detected. An analysis of the COI region showed that the Pv210-infected *An. stephensi* belonged to the dominant invasive haplotypes circulating in the Horn of Africa.

**Conclusions:**

The findings from this study confirm the involvement of *An. stephensi* in P. vivax transmission in Djibouti and the genetic relatedness of Djiboutian *An. stephensi* populations to other populations across the Horn of Africa. This highlights the threat of *An. stephensi* invasion and supports a rapid and comprehensive response, particularly through surveillance and control.

**Graphical Abstract:**

**Figure:**
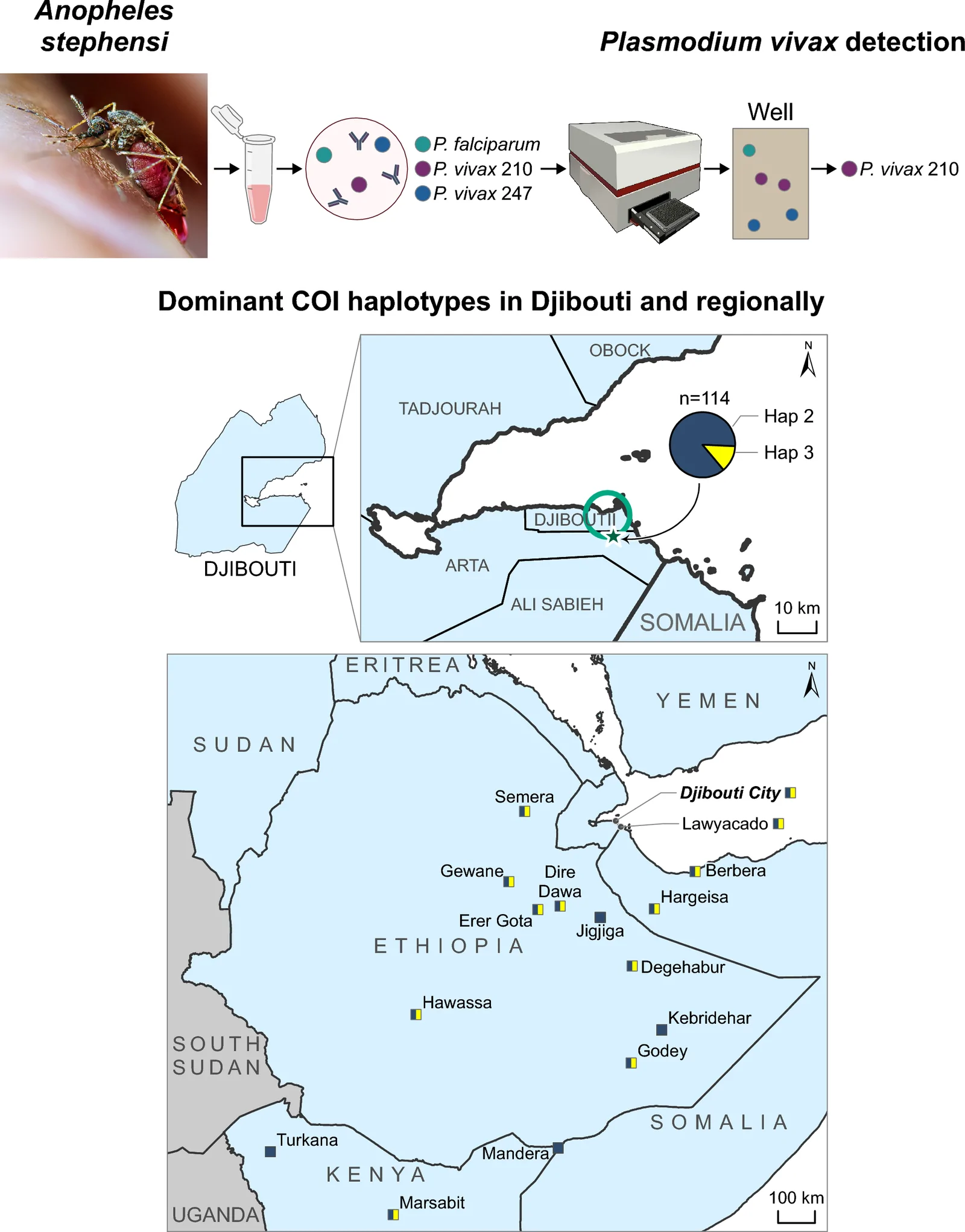

**Supplementary Information:**

The online version contains supplementary material available at https://doi.org/10.1186/s13071-026-07532-9.

## Background

Malaria presents a significant burden to health systems globally, with 263 million cases and over 597,000 deaths reported in 2023, 94% of which occurs in sub-Saharan Africa [1]. Though there are malaria control programs in Djibouti, achieving control and elimination has become increasingly challenging due to emerging threats such as insecticide resistance and invasive malaria vectors like *Anopheles stephensi*.

Native to South Asia and parts of the Middle East, and capable of transmitting both *Plasmodium falciparum* and *P. vivax*, *An.* s*tephensi* was first detected in Djibouti in 2012 [2]. In subsequent years it was detected in multiple African countries, including Ethiopia, Sudan, Somalia, Nigeria, Ghana, Kenya, Eritrea, and Niger [3].

In Djibouti, since the first detection of *An. stephensi* in 2012, malaria cases have increased more than 20-fold, from 1,684 cases in 2013 to 38,944 cases in 2023 [1]. The unprecedented increase includes both *P. falciparum* and *P. vivax* malaria cases [1]. This is highly concerning given that Djibouti was on the verge of reaching the pre-elimination phase of malaria before 2012 [4]. Also, unlike native African malaria vectors, *An. stephensi* persists year-round due to its ability to breed in artificial containers in both urban and peri-urban settings [5–7]. Indeed, *An. stephensi* has surpassed the native African malaria vector *Anopheles arabiensis* to become the primary vector in Djibouti City [7, 8]. It was found implicated in a resurgence of malaria cases in Djibouti City in 2014 [2] and 2019 [9] through positive infectivity for both *P. falciparum* and *P. vivax* based on a qualitative dipstick assay [10]. *Anopheles stephensi* was also suspected in an outbreak among French military personnel in Djibouti City in 2019 [7].

However, the first direct link of the invasive *An. stephensi* involvement in malaria transmission was reported during a malaria outbreak in an urban setting and during the dry season in the neighboring country, Ethiopia, in 2022 [11]. A multiplex bead assay of the circumsporozoite enzyme-linked immunosorbent assay (csELISA) [12] was used to molecularly confirm the involvement of *An. stephensi* in malaria transmission in Ethiopia. This multiplex bead csELISA assay is the most sensitive and specific *Plasmodium* sporozoite detection assay, based on the gold-standard plate-based csELISA. The multiplex bead csELISA assay has not been utilized in *An. stephensi* surveillance in Djibouti. Prior *Plasmodium* sporozoite detections were conducted in 2014 [2] and 2019 [9] using a qualitative dipstick assay [10]. Rapid assays such as dipstick assays are beneficial for on-the-go testing in the field [13]; however, they are less sensitive and specific [14] than molecular assays such as csELISA and bead assay-based csELISA [12]. With the ongoing increase of malaria cases in Djibouti [2, 9], and the risk of malaria *An. stephensi* presents to an additional 125 million people in Africa [15], it is important to continue *Plasmodium* sporozoite testing of wild caught populations to better understand transmission dynamics and mitigate and control the spread of this invasive vector.

Thus, in this study, we aim to (1) use the csELISA molecular approach to confirm that *An. stephensi* is linked to the ongoing malaria outbreak in Djibouti City, and (2) determine its relatedness to other *An. stephensi* in the Horn of Africa. Defining the ongoing role *An. stephensi* plays in the high rates of malaria transmission in the country will inform targeted vector surveillance and control approaches of this invasive malaria species in Djibouti and the invaded region in Africa.

## Methods

Adult *Anopheles* mosquitoes were collected through backpack aspiration at the Djibouti Nagad Railway Station, a train service connecting Djibouti to Addis Ababa, Ethiopia, during the dry season in February 2024 (Additional file 1: Table S1). Collections were conducted following a report of an *An. stephensi*-mediated malaria outbreak during a dry season in Dire Dawa, Ethiopia [11]. Other sites included routine surveillance at fixed sentinel sites in Djibouti City (Additional file 1: Table S1), the capital city of Djibouti, where over 90% of malaria cases have occurred in recent years [16]. All 196 samples collected were morphologically identified and molecularly confirmed using a portion of the cytochrome c oxidase subunit 1 (COI) marker using the Folmer COI primer set LCO1490F (5ʹ-GGTCAACAAATCATAAAGATATTGG-3ʹ) and HCO2198R (5ʹ-TAAACTTCAGGGTGACCAAAAAATCA-3ʹ) yielding a PCR product of 750 base pairs following previously described protocols [17, 18] (Additional file 1: Text S1). All 196 samples were tested for *Plasmodium* sporozoites [11, 12] (Additional file 1: Text S2). Briefly, mosquito heads–thoraces were separated from legs, wings, and abdomens between the second and third legs from all 196 samples, homogenized and tested for *P. falciparum* (*Pf*), *P. vivax*210 (*Pv*210), and *P. vivax*247 (*Pv*247) following the csELISA protocol [12] (Additional file 1: Text S2). All positive detections were further confirmed by a second boil of the homogenate which was then re-tested for *Pf*, *Pv*210, and *Pv*247 following the same csELISA protocol.

Furthermore, the COI DNA sequences were analyzed to generate population genetic statistics and conduct a phylogenetic analysis to further confirm molecular species identification and determine the level of genetic diversity of the sampled *An. stephensi,* and relatedness to other *An. stephensi* populations in the Horn of Africa (Additional file 1: Fig. S1). Briefly, COI sequences were first cleaned using CodonCode version 11.0.2 (CodonCode Corporation, Centerville, MA). Beyond the initial cleaning, 114 out of 196 COI sequences displayed high-quality alignment with previously identified *An. stephensi* haplotypes and were thus appropriate for further comparative analysis between the studied samples and *An. stephensi* populations in the Horn of Africa [5, 19–22]. The extra sequence quality control is important as high-quality DNA sequences are essential for accurate comparative population genetic analysis. Population genetics statistics were generated in DNAsp version 6 [23], including the number of polymorphic (segregating) sites (s), number of haplotypes (h), haplotype diversity (Hd), and nucleotide diversity (π). Next, the COI sequences were further aligned using CodonCode version 11.0.2 (CodonCode Corporation, Centerville, MA) with previously published *An. stephensi* COI sequences from the Horn of Africa retrieved from Genbank. Phylogenetic relationships were inferred using a maximum likelihood approach with RAxML GUI using the GTR model of nucleotide substitutions, gamma model for rate of heterogeneity (GTRGAMMA), and one thousand replicates in one run for bootstrap analysis [24]. *Anopheles maculatus* was designated as an outgroup to be consistent with previous *An. stephensi* phylogenetic analysis [5, 25]. The tree with the highest log likelihood was visualized and formatted using FigTree v1.44 [26]. Lastly, a Horn of Africa map showing the distribution of *An. stephensi* most prevalent COI haplotypes (i.e., Hap 2, and Hap 3) was generated including data from Carter et al. [19], Samake et al. [5], Hawaria et al. [21] (Ethiopia), Ali et al. [20] (Somalia), and Samake et al. [22] (Kenya).

## Results and discussion

**Fig. 1:**
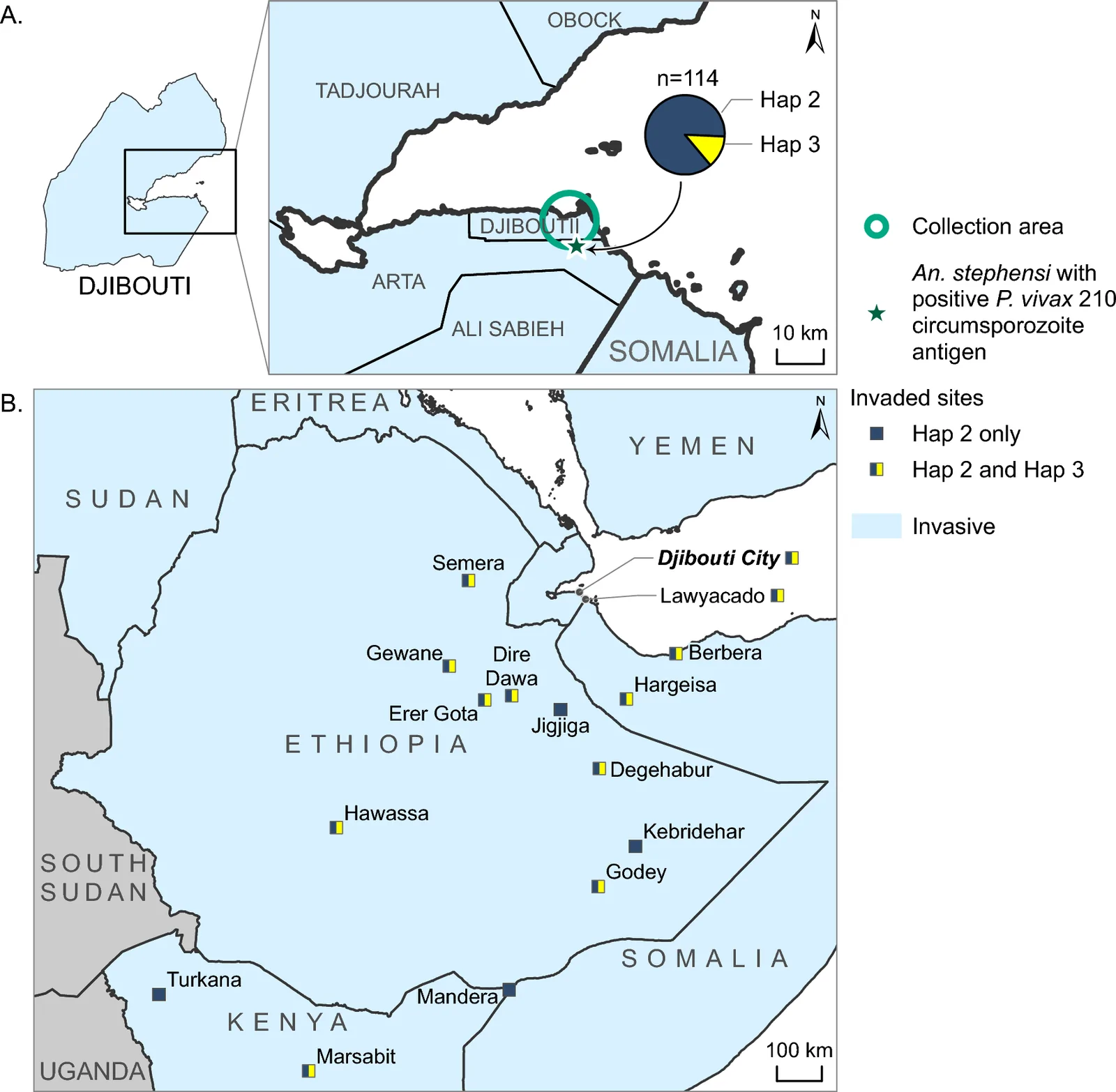
**A** Map of collection area, *Pv*210-positive Djiboutian *An. stephensi* site and COI haplotypes. **B** Map of *An. stephensi* COI haplotype 2 and haplotype 3 distribution across the Horn of Africa. Horn of Africa map includes COI data from previously published reports [5, 19–22]. Maps were generated from administrative boundaries: GADM 4.1 using ArcGIS Pro v 3.5 (Environmental Systems Research Institute (ESRI), Redlands, CA, USA)

Using the NCBI BLAST tool, 196 COI sequences matched with 100% percent identity to *An. stephensi*. Two out of the 196 samples (1.0%) tested positive for the presence of the *Pv*210 circumsporozoite protein, which indicates the presence of malaria in Djiboutian *An. stephensi* (Table 1). Zero out of the 196 samples tested positive for the presence of the *Pv*247 or *Pf* circumsporozoite proteins. The genetic diversity statistics based on 114 out of the 196 generated COI DNA sequences revealed two segregating sites leading to two haplotypes (Table 2). Based on the phylogenetic analysis, the two haplotypes clustered with previously reported *An. stephensi* COI haplotypes in Djibouti, namely, haplotype 2 (*n* = 99) and haplotype 3 (*n* = 15) [27] (Additional file 1: Fig. S1). These two haplotypes were reported as the most widespread COI haplotypes of the invasive *An. stephensi* in Djibouti and across the Horn of Africa [19, 27] (Fig. 1).

**Table 1:** Sample information and infectivity rate

Collection site	Total samples collected	Confirmed as *An. stephensi* through molecular species ID	*P. falciparum*-positive samples (infectivity rate)	*P. vivax* 210-positive samples (infectivity rate)	*P. vivax* 247-positive samples (infectivity rate)
Djibouti City	196	196	0 (0%)	2 (1.0%)	0 (0%)

**Table 2:** Population genetic diversity based on mtDNA COI loci of *Anopheles stephensi* from Djibouti, 2024

Collection site	Sample size (N)	Number of polymorphic (segregating) sites (S)	Number of haplotypes (h)	Haplotype diversity, (Hd)	Nucleotide diversity (π)
Djibouti City	114	2	2	0.231	0.00145

**Fig. 2:**
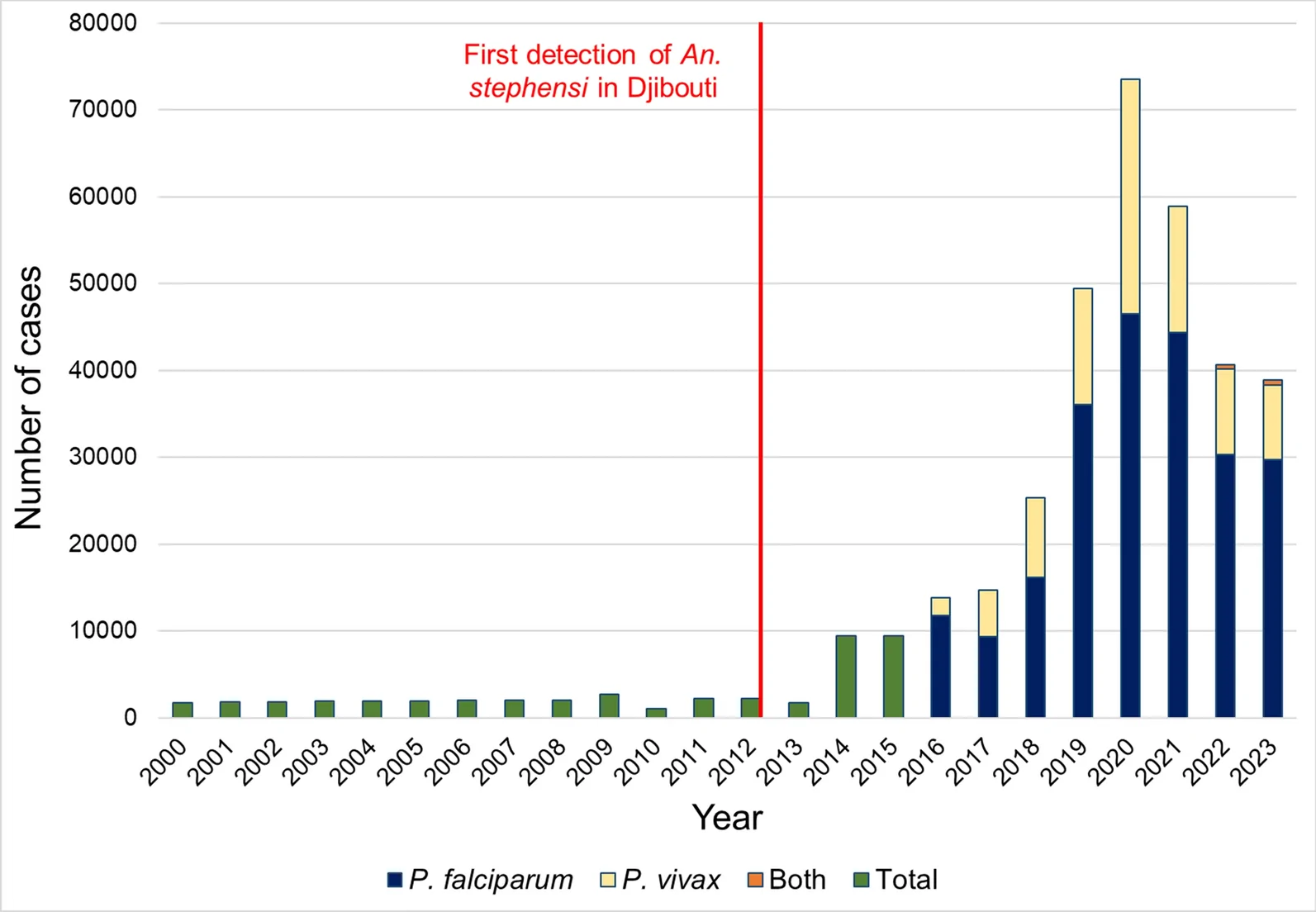
Yearly cases of malaria in Djibouti, 2000–2023. Determination of the *Plasmodium* species and strains began in 2016. Data from World Malaria Reports [1, 28]

The detection of *Pv*210 circumsporozoite protein using the most advanced csELISA approach in a Djiboutian *An. stephensi* population provides highly sensitive and specific molecular evidence for the direct link between *An. stephensi* and malaria transmission in Djibouti. Our finding supports the previous report of *P. vivax* detection in *An. stephensi* in Djibouti City seven years prior [9] and suggests that *An. stephensi* is continuing to play a role in increasingly high incidence rates of *P. vivax* malaria observed in Djibouti in addition to *P. falciparum* malaria [1, 28] (Fig. 2). Indeed, *P. vivax* makes up an average of 28% of malaria cases in Djibouti from 2016–2023 (Fig. 2). Though less prevalent than *P. falciparum*, *P. vivax* constitutes a significant burden on health systems [29]. Based on our findings that invasive *An. stephensi* is contributing to *P. vivax* transmission in Djibouti, it is important to optimize vector control measures to fit the *Plasmodium* species profile of Djibouti and prevent malaria cases overall. Though *P. falciparum* was not detected, which does not reflect the relative cases of *P. vivax* to *P. falciparum* in Djibouti (Fig. 2), it is likely a facet of the utilization of a relatively small (*n* = 196) sample size. Additionally, the fact that these *Plasmodium*-carrying *An. stephensi* were also observed to be closely related to *An. stephensi* populations across the Horn of Africa suggests that the invasive mosquito is possibly contributing to the transmission of malaria in other invaded areas across the region as well (Fig. 1).

Taken together, our findings fill critical gaps by (1) providing information on the detection of circumsporozoite-positive *An. stephensi* in Djibouti given that the last detection occurred seven years ago [9]; (2) providing the first molecularly confirmed circumsporozoite-positive *An. stephensi* using the innovative multiplex bead-based csELISA approach [12]; and (3) characterizing the genetic diversity of a recent *An. stephensi* population in Djibouti and its relatedness to other *An. stephensi* populations in the Horn of Africa.

With the evidence of the continued involvement of *An. stephensi* in malaria transmission in Djibouti, the conventional vector control measures will need adjustments to account for *An. stephensi* distinct behaviors. As *An. stephensi* have been reported to display outdoor biting behaviors, the use of indoor residual spraying (IRS) and insecticide-treated nets (ITNs) alone is insufficient to protect people from bites [30]. Additionally, Djibouti’s coastal location with frequent rainfall and flooding can contribute to an increase in larval habitats [8, 31]. Larval source management (LSM) can combat this by either killing larvae or modifying their habitats to contain the spread of populations [32]. However, LSM does not provide information on transmission indicators, as the circumsporozoite protein can only be detected in adult mosquitoes. Based on the limitations of conventional vector control strategies such as IRS, ITNs, and LSM, additional methods that track entomological indicators such as increasing adult surveillance, would be beneficial for Djibouti. Controlling adults will control all mosquito life stages and help stop further spread of this invasive vector.

## Conclusions

Through this study, we molecularly confirmed that invasive *An. stephensi* is transmitting malaria parasites (*P. vivax* 210) in Djibouti. We additionally confirmed through a COI region analysis that *An. stephensi* populations in Djibouti are closely related to the majority of *An. stephensi* populations found in the Horn of Africa. This highlights the threat of *An. stephensi* invasion and supports a rapid and targeted response to mitigate the harm that *An. stephensi* cause, particularly through adult surveillance and control.

## Supplementary Information

Additional file 1: Table S1. Table of coordinates and elevation of collection sites. **Text S1.** Molecular analysis. **Figure S1.** Phylogenetic tree of Horn of Africa *An. stephensi* cytochrome oxidase subunit I. **Text S2.** Multiplex csELISA Assay.

## Data Availability

The sequences generated in this study are available through NCBI Genbank Accession numbers PX682928–PX683039.

## Abbreviations

*An. stephensi*: *Anopheles stephensi*
BLAST: Basic Local Alignment Search Tool
csELISA: Circumsporozoite enzyme-linked immunosorbent assay
COI: Cytochrome c oxidase subunit 1
DNA: Deoxyribonucleic acid
GTR model: General time reversible model
IRS: Indoor residual spraying
ITN: Insecticide-treated net
LSM: Larval source management
NCBI: National Center for Biotechnology Information
*P. falciparum*: *Plasmodium falciparum*
*P.** vivax*: *Plasmodium vivax*
*Pv*210: *Plasmodium vivax* 210
*Pv*247: *Plasmodium vivax* 247
PCR: Polymerase chain reaction
