# Supplementary material for: Evidence of Anopheles stephensi involvement in the transmission of Plasmodium vivax in Djibouti City using highly sensitive sporozoite detection assay
Source: Parasit Vectors. 2026 Jul 8;19:424. doi: 10.1186/s13071-026-07532-9 (PMC13632229; doi:10.1186/s13071-026-07532-9)
Supplement: Supplementary file 1 [file 13071_2026_7532_MOESM1_ESM.pdf]

Evidence of *Anopheles stephensi* involvement in the transmission of *Plasmodium vivax* in Djibouti City using highly sensitive sporozoite detection assay

Subhika Rao[1], Jeanne N. Samake[1*], Cristina Rafferty[1], Peter Mumba [2], Sheleme Chibsa [2], Meshesha Balkew [3], Bouh Abdi Khaireh [4,5], Samatar Kayad Guelleh [6], Mohamed Mousse Ibrahim [4], Abdoulilah Ahmed Abdi [8], Sarah Zohdy[1]

*correspondence: Jeanne N. Samake lyg2@cdc.gov

Supplementary information

Table S1. Table of coordinates and elevation of collection sites

Site	Neighborhood	Region	Latitude	Longitude
site 1	Arhiba	Djibouti	11°34'47.5"N	43°08'10.6"E
site 7	Chieck Moussa	Djibouti	11°32'26.6"N	43°04'54.5"E
site 7		Djibouti	11°33'01.1"N	43°07'01.8"E
site 7		Djibouti	11°33'07.0"N	43°07'08.1"E
site 6	Cheick Osman	Djibouti	11°33'11.7"N	43°07'18.5"E
site 7		Djibouti	11°33'03.2"N	43°07'00.3"E
site 7		Djibouti	11°33'04.4"N	43°07'01.2"E
site 5	Cite Nassib	Djibouti	11°32'03.5"N	43°05'01.2"E
site 5		Djibouti	11°32'24.1"N	43°05'01.5"E
site 5		Djibouti	11°32'26.5"N	43°04'54.7"E
site 5		Djibouti	11°32'15.9"N	43°04'35.0"E
site 5		Djibouti	11°32'30.0"N	43°04'56.2"E
site 5		Djibouti	11°32'57.3"N	43°05'14.0"E
site 5		Djibouti	11°32'02.3"N	43°04'47.2"E
site 5		Djibouti	11°32'07.9"N	43°04'45.9"E
site 5		Arta	11°31'50.9"N	43°04'54.5"E
site 5		Arta	11°31'52.1"N	43°04'39.6"E
site 5		Djibouti	11°32'29.5"N	43°04'33.9"E
site 5		Djibouti	11°32'20.0"N	43°04'43.1"E
site 2	Cite Stade	Djibouti	11°33'40.6"N	43°08'27.3"E
site 2		Djibouti	11°34'32.7"N	43°08'46.5"E
site 2		Djibouti	11°33'36.1"N	43°08'54.6"E
site 1	FNP	Djibouti	11°35'02.0"N	43°08'27.2"E
site 2	Gabod 2	Djibouti	11°33'48.5"N	43°08'49.1"E
site 2		Djibouti	11°34'29.3"N	43°09'01.3"E
site 2		Djibouti	11°34'32.7"N	43°08'46.5"E
site 2	Gabod 4	Djibouti	11°34'30.2"N	43°09'29.2"E

**Site**	**Neighborhood**	**Region**	**Latitude**	**Longitude**
site 2		Djibouti	11°33'58.0"N	43°08'51.5"E
site 2		Djibouti	11°33'40.6"N	43°08'27.3"E
site 2		Djibouti	11°34'29.3"N	43°09'01.3"E
site 2		Djibouti	11°33'36.1"N	43°08'54.6"E
site 2	Gabod 5	Djibouti	11°33'48.7"N	43°08'49.4"E
site 2		Djibouti	11°33'58.1"N	43°08'41.2"E
site 2		Djibouti	11°33'48.6"N	43°08'49.7"E
site 2		Djibouti	11°33'59.5"N	43°08'56.7"E
site 2		Djibouti	11°34'30.9"N	43°08'50.6"E
site 3	Héron	Djibouti	11°33'48.8"N	43°08'49.6"E
site 3		Djibouti	11°36'16.8"N	43°09'20.7"E
site 3		Djibouti	11°36'33.2"N	43°09'02.9"E
site 3		Djibouti	11°36'45.6"N	43°08'53.4"E
site 3		Djibouti	11°36'54.1"N	43°08'49.9"E
site 3		Djibouti	11°36'49.8"N	43°08'50.6"E
site 3		Djibouti	11°36'14.3"N	43°09'11.7"E
site 3		Djibouti	11°36'13.5"N	43°09'20.2"E
site 3		Djibouti	11°36'24.5"N	43°09'08.9"E
site 7	Langabaleh	Djibouti	11°33'06.6"N	43°04'28.3"E
site 7		Djibouti	11°33'10.9"N	43°07'10.8"E
site 7		Djibouti	11°32'57.9"N	43°07'07.9"E
site 1	Maka Makarama	Djibouti	11°35'16.6"N	43°08'09.9"E
site 1		Djibouti	11°35'16.4"N	43°08'02.5"E
site 1	Maka Moukaram	Djibouti	11°34'44.4"N	43°08'06.1"E
site 1	Moukaram	Djibouti	11°35'05.1"N	43°08'09.9"E
site 1		Djibouti	11°34'44.4"N	43°08'06.1"E
Arta	Nagad Railway Station	Arta	11°31′24″N	43°07′39″E
site 2	Plateau	Djibouti	11°36'13.5"N	43°09'20.2"E
site 3	Plateau Siesta	Djibouti	11°36'56.8"N	43°08'50.9"E
site 4	PK12	Djibouti	11°32'23.8"N	43°04'50.2"E
site 4		Djibouti	11°33'20.5"N	43°04'55.9"E
site 4		Djibouti	11°33'03.1"N	43°05'28.9"E
site 4		Djibouti	11°33'12.3"N	43°04'59.0"E
site 4		Djibouti	11°33'29.3"N	43°04'55.9"E
site 4		Djibouti	11°33'31.4"N	43°04'31.7"E
site 4		Djibouti	11°33'32.3"N	43°05'33.1"E
site 4		Djibouti	11°33'38.9"N	43°04'51.6"E
site 4		Djibouti	11°33'38.9"N	43°04'50.7"E

Site	Neighborhood	Region	Latitude	Longitude
site 4		Djibouti	11°33'31.6"N	43°04'50.5"E
site 4	PK13	Djibouti	11°32'24.2"N	43°05'00.2"E
site 4		Djibouti	11°34'22.1"N	43°05'04.3"E
site 4		Djibouti	11°33'48.8"N	43°05'07.1"E
site 4		Djibouti	11°34'24.6"N	43°05'07.1"E
site 4		Djibouti	11°34'04.3"N	43°05'29.0"E
site 4		Djibouti	11°34'04.2"N	43°05'37.9"E
site 4	PK13/Ouma Salam	Djibouti	11°34'35.5"N	43°05'09.4"E
site 4	PK14	Djibouti	11°32'18.2"N	43°04'59.6"E
site 6	Q2	Djibouti	11°35'27.4"N	43°08'57.9"E
site 6	Q3	Djibouti	11°35'17.0"N	43°09'03.6"E
site 6		Djibouti	11°35'02.9"N	43°09'03.0"E
site 6	Q4	Djibouti	11°35'12.3"N	43°08'25.5"E
site 6		Djibouti	11°35'14.9"N	43°09'04.2"E
site 6		Djibouti	11°35'11.7"N	43°09'02.8"E
site 6		Djibouti	11°35'00.3"N	43°09'03.9"E
site 6	Q5	Djibouti	11°35'02.9"N	43°09'03.4"E
site 6		Djibouti	11°34'54.2"N	43°08'53.0"E
site 6		Djibouti	11°35'03.1"N	43°08'57.4"E
site 6		Djibouti	11°35'11.7"N	43°09'02.9"E
site 6		Djibouti	11°35'00.3"N	43°09'03.9"E
site 6	Q6	Djibouti	11°35'03.1"N	43°08'57.4"E
site 6		Djibouti	11°34'54.1"N	43°08'54.8"E
site 6		Djibouti	11°34'57.3"N	43°08'53.6"E
site 1	Saline d'Ouest	Djibouti	11°35'16.4"N	43°08'02.5"E
site 1		Djibouti	11°35'09.1"N	43°07'53.6"E
site 1		Djibouti	11°35'03.2"N	43°08'09.1"E
site 1		Djibouti	11°35'05.1"N	43°08'09.9"E
site 1		Djibouti	11°34'47.5"N	43°08'10.6"E
site 1		Djibouti	11°35'04.1"N	43°08'06.1"E
site 1		Djibouti	11°35'03.3"N	43°08'08.5"E
site 1	Wadajira	Djibouti	11°34'36.4"N	43°07'57.5"E
site 8	Warabaley 1	Djibouti	11°33'59.5"N	43°04'33.4"E
site 8		Djibouti	11°34'20.2"N	43°05'35.6"E
site 8		Djibouti	11°34'04.3"N	43°05'29.0"E
site 8		Djibouti	11°34'01.1"N	43°05'32.2"E
site 8		Djibouti	11°34'22.6"N	43°05'37.9"E
site 8		Djibouti	11°34'05.9"N	43°05'36.5"E

Site	Neighborhood	Region	Latitude	Longitude
site 8		Djibouti	11°34'05.5"N	43°05'29.7"E
site 3	Zone Portiaire	Djibouti	11°36'57.7"N	43°08'50.8"E
site 7	55 Logement	Djibouti	11°33'03.1"N	43°06'59.9"E
site 7		Djibouti	11°32'57.9"N	43°07'07.9"E
site 7		Djibouti	11°33'07.0"N	43°07'01.2"E

Text S1. Molecular analysis.

DNA was extracted from the legs using Qiagen DNeasy Blood and Tissue kit (Qiagen, Hilden, Germany). Portions of the COI loci were PCR amplified using the Folmer COI primer set LCO1490F (5′-GGTCAACAAATCATAAAGATATTGG-3′) and HCO2198R (5′-TAAACTTCAGGGTGACCAAAAAATCA-3′) yielding a PCR product of 750 base pairs [1] and following previously described protocols [2]. The COI PCR products were sequenced using Sanger sequencing technology. Sequences were analyzed and cleaned using CodonCode Aligner version 11.0.2 (CodonCode Corporation, Centerville, MA) then queried to NCBI BLAST to confirm correct *An. stephensi* COI locus. Next, population genetic statistics were generated in DNAsp version 6 [3], including the number of polymorphic (segregating) sites (s), number of haplotypes (h), haplotype diversity (Hd), and nucleotide diversity (π). Lastly, the COI sequences were further aligned in CodonCode with previously published *An. stephensi* COI sequences from the Horn of Africa retrieved from Genbank. Phylogenetic relationships were inferred using a maximum likelihood approach with RAxML GUI using the GTR model of nucleotide substitutions, gamma model for rate of heterogeneity (GTRGAMMA), and one thousand replicates in one run for bootstrap analysis [4]. The tree with the highest log likelihood was visualized and formatted using FigTree v1.44 [5].

Additionally, a Horn of Africa map showing the distribution of *An. stephensi* most prevalent COI haplotypes (i.e., Hap 2, and Hap 3) was generated including data from Carter et al. [6], Samake et al. [7], Hawaria et al. [8] (Ethiopia), Ali et al. [9] (Somalia), and Samake et al. [10](Kenya).

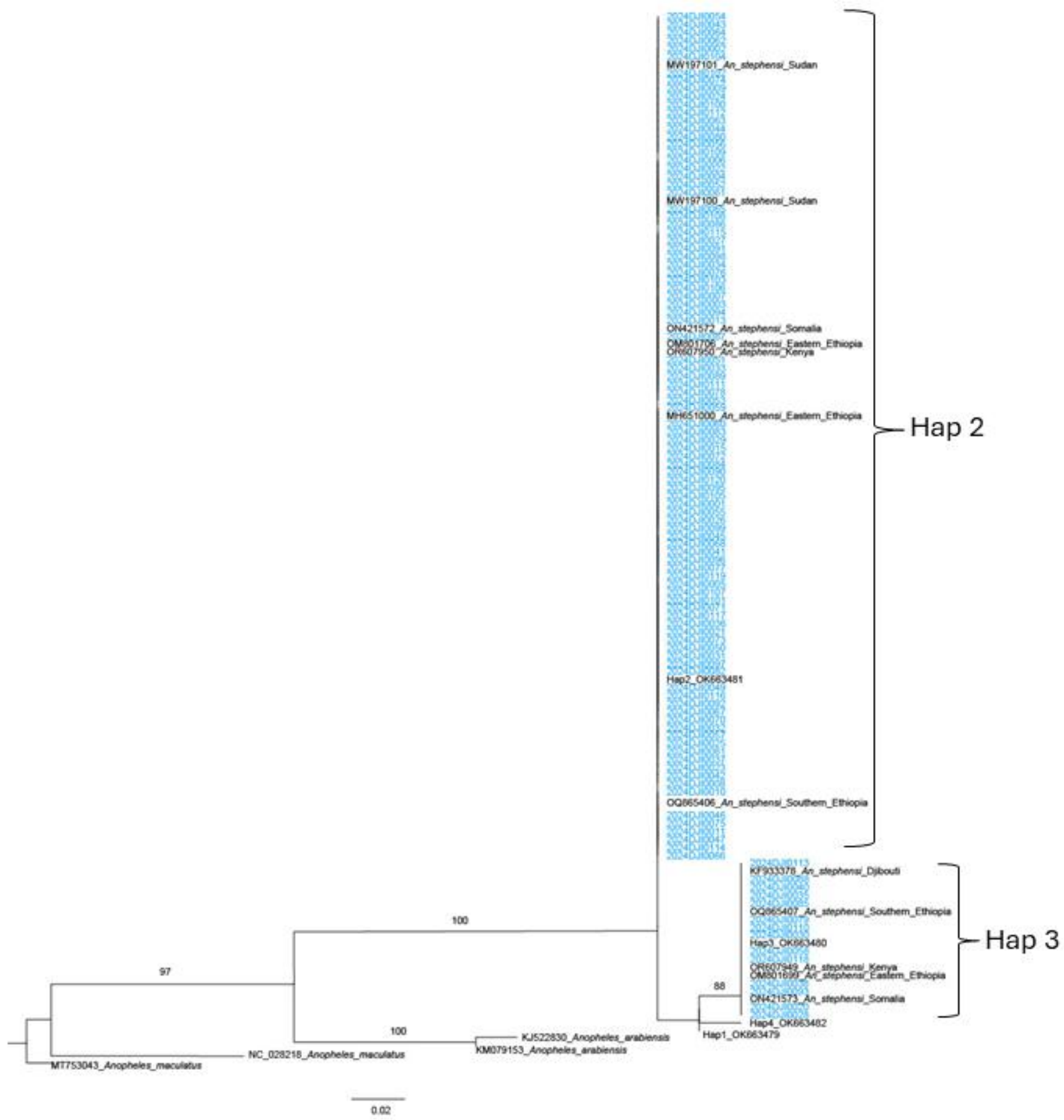

Fig. S1. Phylogenetic tree of Horn of Africa *An. stephensi* cytochrome oxidase subunit I (COI). The evolutionary history was inferred by using the Maximum Likelihood method based on the General Time Reversible model. Blue = Djibouti COI data generated in this study. Numbers on the branches indicate bootstrap values (only values > 70 are shown). Scale bar indicates the number of nucleotide substitutions per site. Haplotype numbering was based on haplotypes list from Carter et al. [6].

Text S2. Multiplex csELISA Assay.

We followed the protocol as published in Sutcliffe et al. [11]. Following single-plex reactions for assay optimization, the bead assay was performed in multiplex containing *P. falciparum*, *P. vivax*210 and *P. vivax*247 coupled beads and detection antibodies. Reagent diluent (0.45 µM filter-sterilized PBS-T, 0.5% BSA) was used to dilute bead-coupled antibody, biotinylated detection antibody and streptavidin–phycoerythrin (Invitrogen; 2866). In each step where reagent diluent was used, 50 µl of solution was applied per well. Incubations were carried out, protected from light, at room temperature (approximately 20 °C) using a plate shaker (IKA; MTS 2/4) at 900 rpm and three wash steps with PBS-T were performed between each incubation. For the assay, filter bottom plates (Millipore; MADVN6550) were prewetted with PBS-T and 50 µl

(approximately 1250 beads) of coupled beads were added, washed three times, and incubated with 50 µl of sample for 1.5 h. After washing, wells were incubated with a 50 µl mixture of *P. falciparum*, *P. vivax*210 and *P. vivax*247 biotinylated detection antibodies for 45 min. After washing, wells had a 30-min incubation with 50 µl of streptavidin–phycoerythrin in reagent diluent (Invitrogen; 2866). Wells had a final incubation with reagent diluent for 30 min before resuspension in 100 µl of PBS for a brief (1–2 min) incubation and were then either analysed immediately using a Bio-Plex® 200 system (BioRad; 171000201) and Bio-Plex® Manager™ software v6.2 (Bio-Rad, USA) with a target of 50 beads per region or stored at 4 °C, protected from light, for up to 24 h. Stored plates were washed three times following removal of PBS, wells were resuspended in 100 µl of PBS using a brief (1–2 min) incubation and then analysed as described above [12]. The Bio-Plex® 200 system detects emitted fluorescence from the microspheres and the biotinylated detection antibody. Bio-Plex® Manager™ software reports fluorescence as the median fluorescence intensity (MFI) and generates the mean MFI for replicates when applicable. Background (Bkgd) values calculated from sample wells containing csELISA grind buffer alone were subtracted from MFI values to report the final assay signal as MFI-Bkgd.

References

1. Folmer O, Black M, Hoeh W, Lutz R, Vrijenhoek R. DNA primers for amplification of mitochondrial cytochrome c oxidase subunit I from diverse metazoan invertebrates. Mol Mar Biol Biotechnol. 1994;3(5):294-9.
2. Waymire E, Samake JN, Gunarathna I, Carter TE. A decade of invasive *Anopheles stephensi* sequence-based identification: toward a global standard. Trends Parasitol. 2024;40(6):477-86.
3. Rozas J, Ferrer-Mata A, Sánchez-DelBarrio JC, Guirao-Rico S, Librado P, Ramos-Onsins SE, et al. DnaSP 6: DNA Sequence Polymorphism Analysis of Large Data Sets. Mol Biol Evol. 2017;34(12):3299-302.
4. Stamatakis A. RAxML version 8: a tool for phylogenetic analysis and post-analysis of large phylogenies. Bioinformatics. 2014;30(9):1312-3.
5. Rambaut A. Figtree ver 1.4.4. Institute of Evolutionary Biology, University of Edinburgh, Edinburgh. 2018.
6. Carter TE, Yared S, Getachew D, Spear J, Choi SH, Samake JN, et al. Genetic diversity of *Anopheles stephensi* in Ethiopia provides insight into patterns of spread. Parasit Vectors. 2021;14(1):602.
7. Samake JN, Yared S, Hassen MA, Zohdy S, Carter TE. Insecticide resistance and population structure of the invasive malaria vector, *Anopheles stephensi*, from Fiq, Ethiopia. Sci Rep. 2024;14(1):27516.
8. Hawaria D, Kibret S, Zhong D, Lee MC, Lelisa K, Bekele B, et al. First report of *Anopheles stephensi* from southern Ethiopia. Malar J. 2023;22(1):373.
9. Ali S, Samake JN, Spear J, Carter TE. Morphological identification and genetic characterization of *Anopheles stephensi* in Somaliland. Parasit Vectors. 2022;15(1):247.
10. Samake JN, Athinya DK, Milanoi S, Ramaita E, Muchoki M, Omondi S, et al. Spatial distribution and population structure of the invasive *Anopheles stephensi* in Kenya from 2022 to 2024. Scientific Reports. 2025;15(1):1-14.
11. Sutcliffe AC, Irish SR, Rogier E, Finney M, Zohdy S, Dotson EM. Adaptation of ELISA detection of *Plasmodium falciparum* and *Plasmodium vivax* circumsporozoite proteins in mosquitoes to a multiplex bead-based immunoassay. Malar J. 2021;20(1):377.
12. Zhang Y, Li X, Di YP. Fast and Efficient Measurement of Clinical and Biological Samples Using Immunoassay-Based Multiplexing Systems. Methods Mol Biol. 2020;2102:129-47.
